# Free-hand technique of C7 pedicle screw insertion using a simply defined entry point without fluoroscopic guidance for cervical spondylotic myelopathy patients with C3 to C6 instrumented by lateral mass screws: a retrospective cohort study

**DOI:** 10.1186/s12893-024-02358-7

**Published:** 2024-02-29

**Authors:** Jun Jiang, Chen-yu Song, Zheng-zheng Wu, Zuo-zhi Xie, Bo Shi, Tao Xu, Han Wang, Yong Qiu, Bin Wang, Ze-zhang Zhu, Yang Yu

**Affiliations:** 1 Division of Spine Surgery, Department of Orthopedic Surgery, Nanjing Drum Tower Hospital, The Affiliated Hospital of Nanjing University Medical School, Nanjing, China; 2 Department of Radiology, Nanjing Drum Tower Hospital, The Affiliated Hospital of Nanjing University Medical School, Nanjing, China

**Keywords:** Cervical, Pedicle screw, Trajectory, Computed tomography

## Abstract

**Background:**

Nowadays, both lateral mass screw (LMS) and pedicle screw were effective instrumentation for posterior stabilization of cervical spine. This study aims to evaluate the feasibility of a new free-hand technique of C7 pedicle screw insertion without fluoroscopic guidance for cervical spondylotic myelopathy (CSM) patients with C3 to C6 instrumented by lateral mass screws.

**Methods:**

A total of 53 CSM patients underwent lateral mass screws instrumentation at C3 to C6 levels and pedicle screw instrumentation at C7 level were included. The preoperative 3-dimenional computed tomography (CT) reconstruction images of cervical spine were used to determine 2 different C7 pedicle screw trajectories. Trajectory A passed through the axis of the C7 pedicle while trajectory B selected the midpoint of the base of C7 superior facet as the entry point. All these 53 patients had the C7 pedicle screw inserted through trajectory B by free-hand without fluoroscopic guidance and the postoperative CT images were obtained to evaluate the accuracy of C7 pedicle screw insertion.

**Results:**

Trajectory B had smaller transverse angle, smaller screw length, and smaller screw width but both similar sagittal angle and similar pedicle height when compared with trajectory A. A total of 106 pedicle screws were inserted at C7 through trajectory B and only 8 screws were displaced with the accuracy of screw placement as high as 92.5%.

**Conclusion:**

In CSM patients with C3 to C6 instrumented by LMS, using trajectory B for C7 pedicle screw insertion is easy to both identify the entry point and facilitate the rod insertion.

## Introduction

Nowadays, both lateral mass screw (LMS) and pedicle screw were effective instrumentation for posterior stabilization of cervical spine [1–4]. Cervical pedicle screw had more pull-out strengths and lower risk of loosening when compared with cervical lateral mass screw, leading to its increasing usage in the surgical treatments of traumatic or non-traumatic disease of cervical spine [5–7]. However, cervical pedicle screw insertion is technically demanding due to both the small morphology of cervical vertebra and the potential risk of injuries of vital surrounding neurovascular structures, such as vertebral artery and spinal cord [8, 9]. Although several techniques have been developed to increase the safety of cervical pedicle screw insertion, such as navigation assistance and robotic guidance, these techniques are costly and increase both radiation exposure and surgical time [10, 11]. Hence, the LMS is still widely used in posterior cervical surgery since placement of cervical LMS is safe and convenient.

In the subaxial cervical spine, C7 has unique anatomic characteristics compared with C3 to C6 because it is a transitional vertebra from cervical spine to thoracic spine [12, 13]. Usually, the LMS was not recommended in C7 due to the thinness of lateral mass, which could not provide enough holding power. Therefore, pedicle screw is the most suitable fixation for C7.

As we know, several techniques had been developed for insertion of LMS in subaxial cervical spine, such as the Magerl, the Anderson, and the An methods [14]. The entry point of cervical LMS was located near the center of lateral mass, no matter which technique was used. However, the recommended entry points for C7 pedicle screw were often located in the upper lateral part of the lateral mass in the literature. Such inconsistency of the screw entry points could lead to difficulty of rod insertion in patients with C3 to C6 instrumented by LMS and C7 instrumented by pedicle screw. In the current study, we introduced an easily defined entry point for C7 pedicle screw, which was in line with the entry points for LMS, described the anatomic parameters of a new C7 pedicle screw trajectory through this entry point, and evaluated the accuracy of C7 pedicle screw insertion by free-hand using this trajectory.

## Materials and methods

### Subjects

With the approval from the institutional review board in our hospital, cervical spondylotic myelopathy (CSM) patients underwent posterior cervical laminectomy from Sept. 2019 to July 2022 were retrospectively reviewed. The inclusion criteria were: (1) with C7 instrumented by pedicle screw using the midpoint of base of superior facet as the entry point; (2) having both preoperative and postoperative 3-dimenional CT reconstructions of the cervical spine. The exclusion criteria were: (1) with congenital malformation of cervical spine; (2) with a history of infectious or traumatic condition of cervical spine. Finally, there were 53 cases (44 males and 9 females) with an average age of 61.9 years (range, 42 to 86 years) included in this study. Informed consent was obtained from all participants.

### CT measurements

**Fig. 1:**
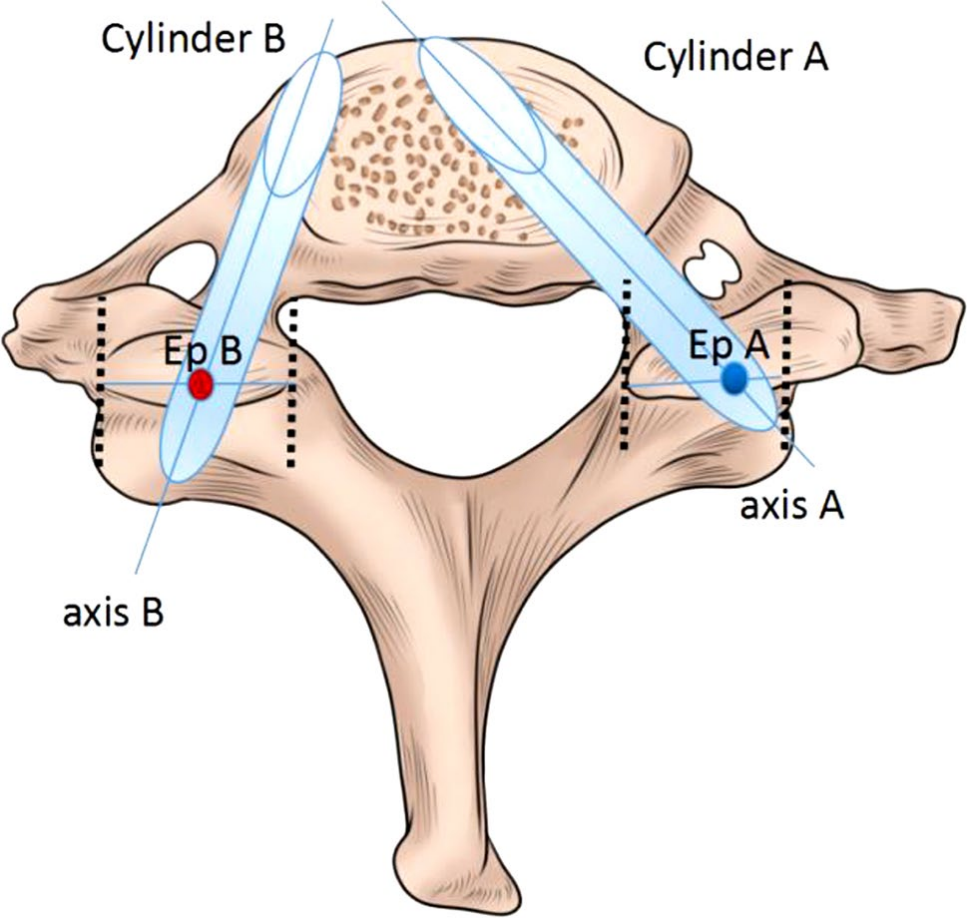
Illustration of two different trajectories for C7 pedicle screw insertion. Axis of cylinder A passed through the center of C7 pedicle while axis of cylinder B penetrated the posterior aspect of C7 at the midpoint of the base of superior facet

**Fig. 2:**
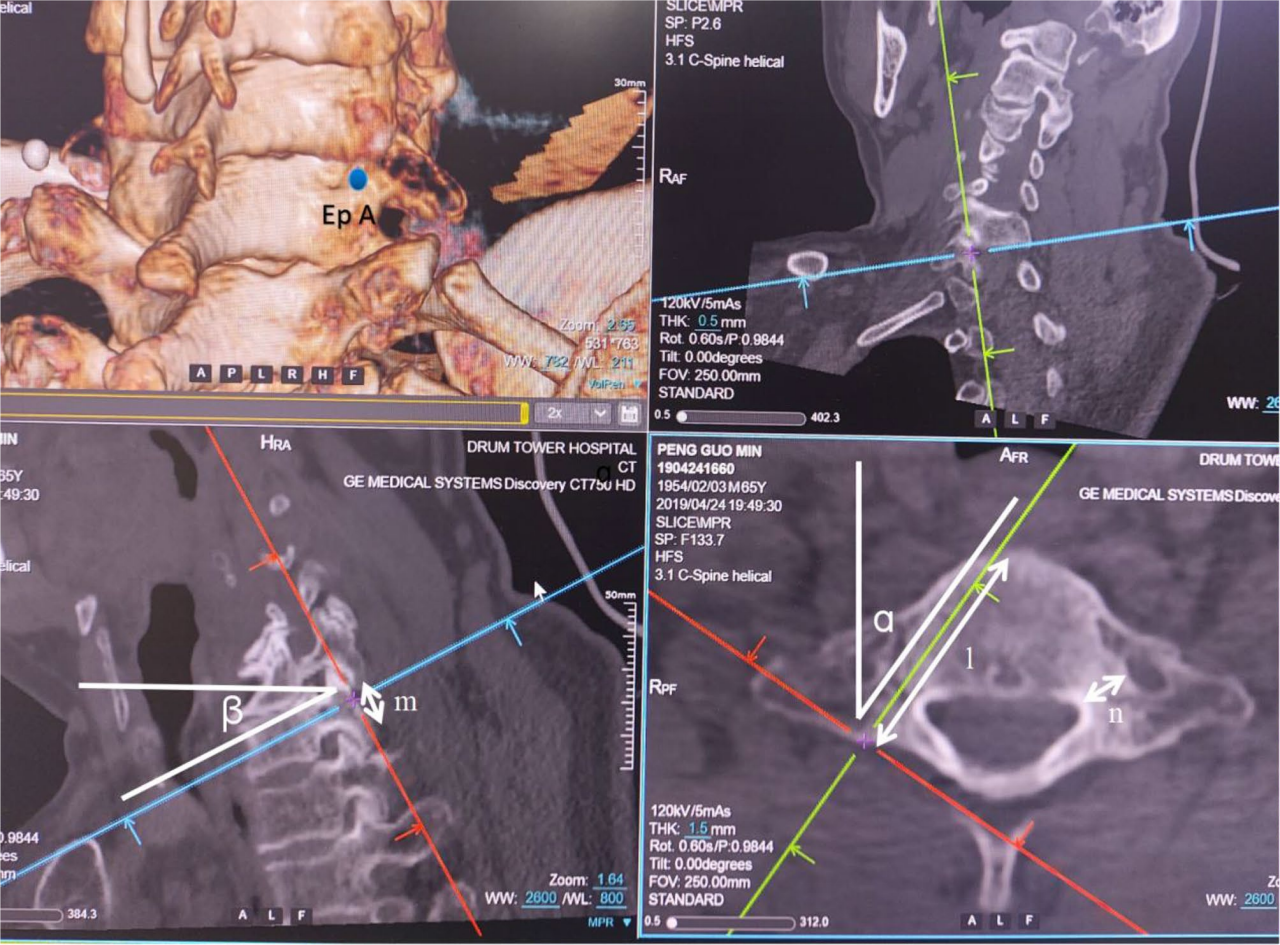
Illustration of trajectory A. Trajectory A passed through the center of C7 pedicle on axial, sagittal and coronal planes. ɑ: transverse angle; β: sagittal angle; l: screw length; m: sagittal height; n: screw width

**Fig. 3:**
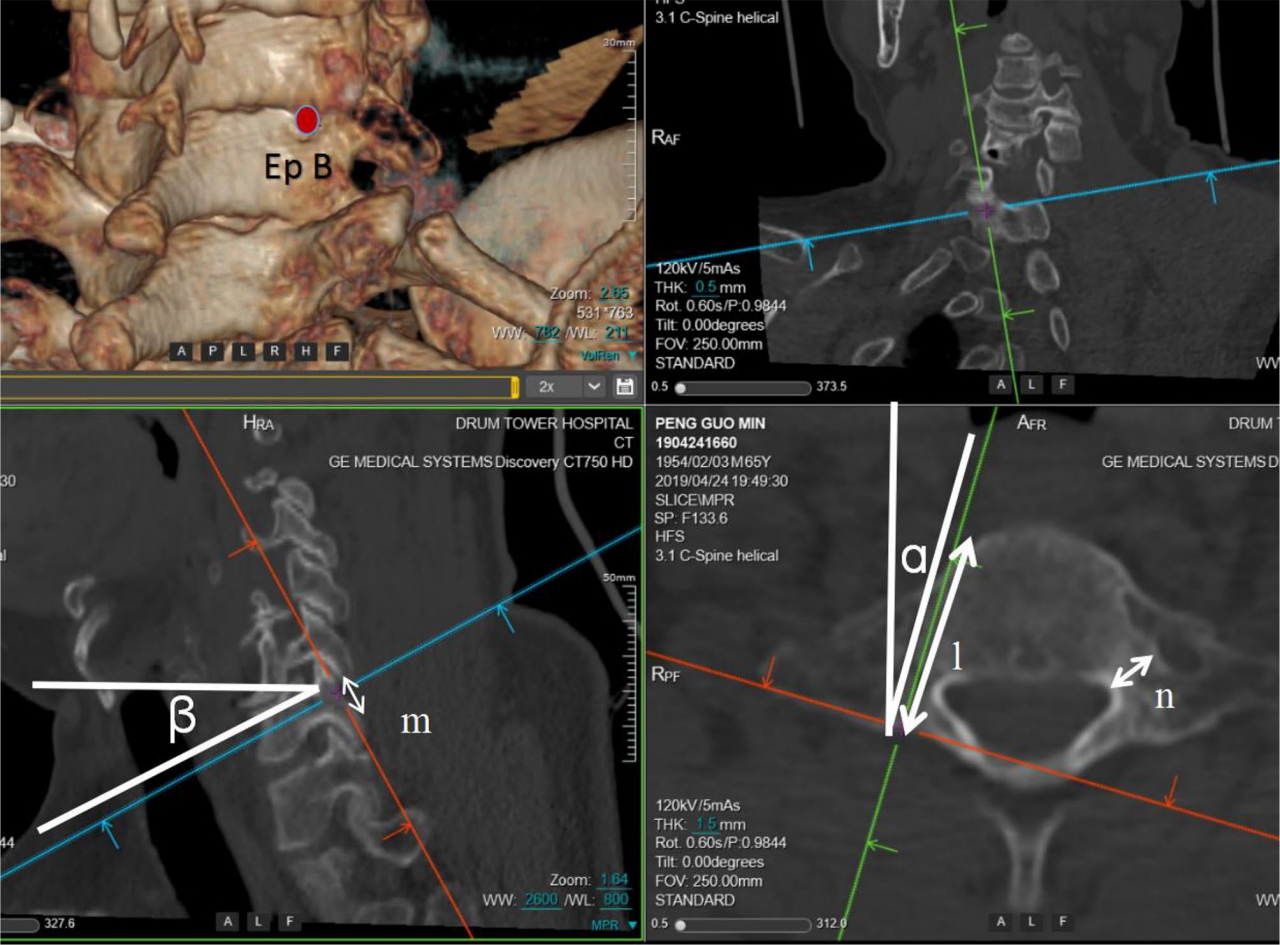
Illustration of trajectory B. Ep B was located at the midpoint of the base of C7 superior facet. ɑ: transverse angle; β: sagittal angle; l: screw length; m: sagittal height; n: screw width

**Fig. 4:**
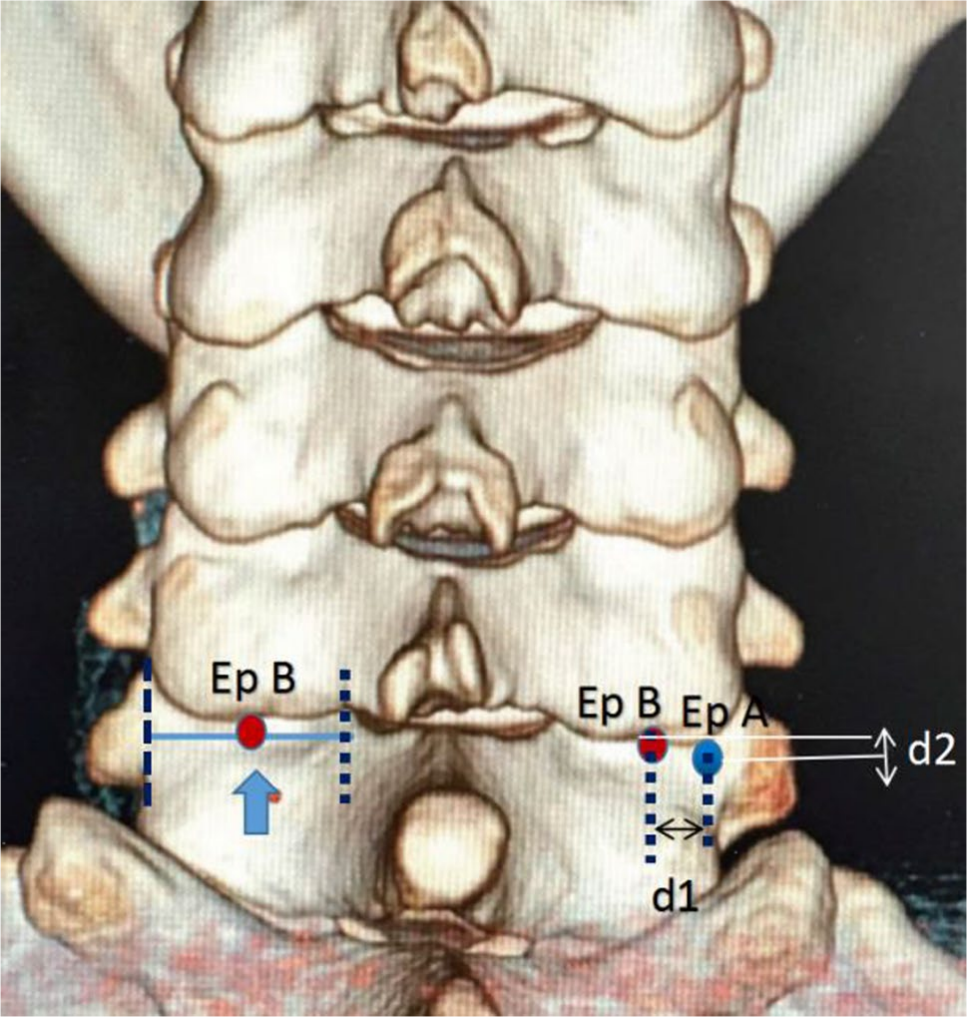
d1: the horizontal distance between Ep A and Ep B; d2: the vertical distance between Ep A and Ep B. The value was positive if Ep A was above Ep B and negative if Ep A was below Ep B

**Fig. 5:**
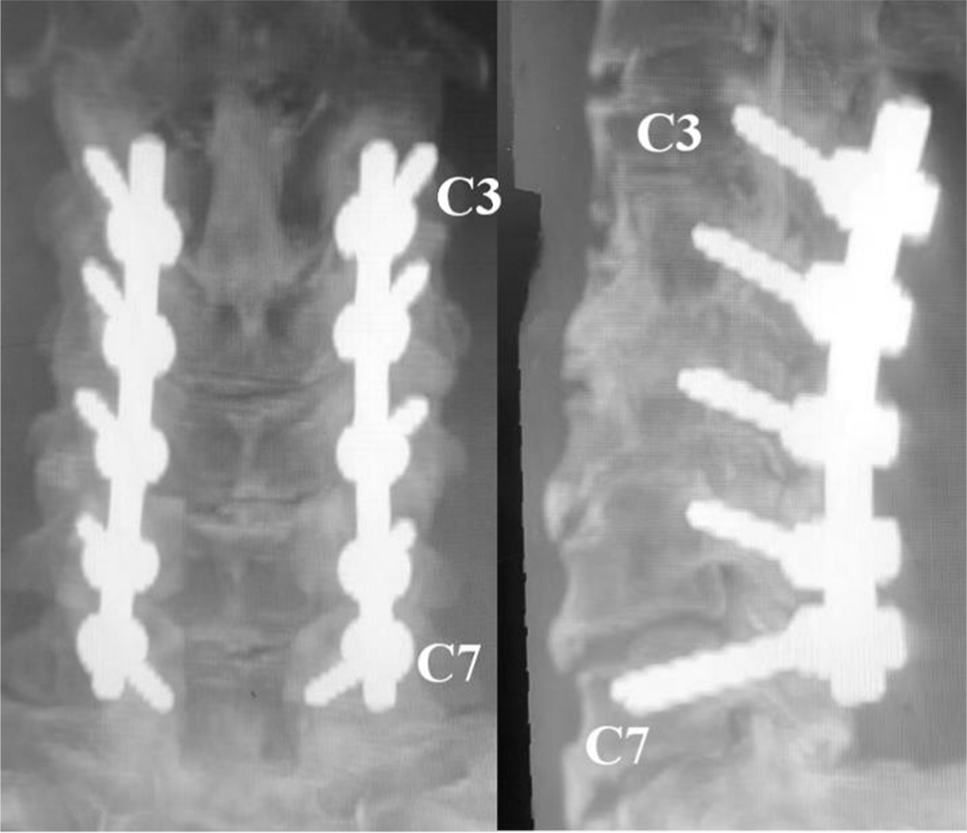
The entry point of C7 pedicle screw insertion through trajectory B was keep in line with the entry points of lateral mass screws from C3 to C6, which facilitated the rod insertion

CT scans of the cervical spine were performed by Brilliance CT 64-channel scanner (Philips Medical Systems, PC Best, Netherlands) with the following parameters: 320 mAs, 120 kvP, 1 mm slice thickness, with a 1 mm gap between slices. These CT images were reconstructed as 3-demenional models using Light speed workplace AW4.3 (General electric company, USA) with matched software. Two different cylinders (A and B) were constructed to simulate the insertion of C7 pedicle screw (Fig. 1). The axis of cylinder A (trajectory A) passed through the center of C7 pedicle on axial, sagittal and coronal planes (Fig. 2). This trajectory penetrated the posterior aspect of lateral mass and the point was identified as the entry point for trajectory A (Ep A). The entry point for axis of cylinder B (trajectory B) was located at the midpoint of the base of C7 superior facet (Ep B) (Fig. 3). Both two cylinders were determined by manipulating the CT imaging planes with both maximal length and maximal width while not violating any of aspect of cortex of the vertebra. (Figures 2 and 3). Five CT parameters of C7 pedicle screw trajectory were measured: (1) transverse angle: the angle between the axis of cylinder and the perpendicular line in the transverse plane (Figs. 2 and 3); (2) sagittal angle: the angle between axis of cylinder and the horizontal line in the sagittal plane (Figs. 2 and 3); (3) screw length: the length of the axis of cylinder from the entry point to the anterior edge of the vertebra (Figs. 2 and 3); (4) screw width: the width of the cylinder (Figs. 2 and 3), (5) pedicle height: distance between superior edge of pedicle and inferior edge of pedicle in the sagittal plane (Figs. 2 and 3), (6) the horizontal distance between Ep A and Ep B (Fig. 4), and (7) the vertical distance between Ep A and Ep B (Fig. 4). The value was positive if Ep A was above Ep B and negative if Ep A was below Ep B.

### Surgical technique

Under general anesthesia, the patient was were placed in a prone position with the head fixed by Mayfield tongs. After the midline incision, the posterior parts of the cervical spine (C3 to C7) were exposed with sub-periosteal dissection to the lateral edge of the lateral mass. The lateral mass screws were inserted from C3 to C6 bilaterally using An technique. The entry point of C7 pedicle screw was located at the midpoint of base of superior facet. After identifying the entry point, a depth of 3–5 mm of pilot hole was drilled using a 2 mm high-speed burr. Then a pathfinder was slightly advanced into the pedicle perpendicularly to the superior spinous ligament with a convergent angle of about 15 degrees. After inserting a depth of 15 mm, the pathfinder was removed and pedicle sound was used to feel the intact cortices of the pedicle. The length of C7 pedicle screw was determined by evaluating the maximal inserted depth of the pedicle sound. After that, a pedicle screw with diameter of either 3.5 or 4.0 mm (based on preoperative CT parameters) and appropriate length was carefully placed. All these C7 pedicle screws were inserted without any fluoroscopy, computer-assisted technique or intraoperative navigation technique to avoid radiation exposure. The procedure of C7 pedicle screw insertion was monitored with both somatosensory-evoked and motor-evoked potentials.

### Accuracy of C7 pedicle screw insertion

Postoperative CT scan of cervical spine was performed in all the patients. The perforations of the bony cortex by C7 pedicle screw were measured in millimeters and were divided into 5 grades: grade 0 (fully contained within the pedicle), grade 1 (perforation ≤ 2 mm), grade 2 (perforation 2.1–4.0 mm), grade 3 (perforation 4.1–6.0 mm) and grade 4 (perforation 6.1–8.0 mm).

### Statistical analysis

Statistical analysis was performed by SPSS software for Windows (16.0, Chicago, IL). All the CT parameters were compared between two different C7 pedicle screw trajectories by independent-t test. The difference of accuracy of screw placement between left side and right side was compared by chi-square test. Significance was established at the *P* < 0.05 level.

## Results

Trajectory B had smaller transverse angle (L:15.3° vs. 33.6°; R:14.4° vs. 32.0°, *P* < 0.001), smaller screw length (L:25.5 mm vs. 32.9 mm; R: 24.7 mm vs. 32.1 mm, *P* < 0.001), and smaller screw width (L:4.6 mm vs. 5.5 mm ;R:4.4 mm vs. 5.5 mm, *P* < 0.001) but both similar sagittal angle (L:32.7° vs. 32.4°; R:32.5° vs. 32.8°, *P* > 0.05) and similar pedicle height (L:5.4 mm vs. 5.6 mm; R: 5.5 mm vs. 5.5 mm, *P* > 0.05) when compared with trajectory A (Table 1). The average horizontal distance between Ep A and Ep B was 3.7 mm on the left side and 3.6 mm on the right side. The average vertical distance between Ep A and Ep B was − 0.7 mm on the left side and − 0.8 mm on the right side.

A total of 106 pedicle screws were inserted at C7 through trajectory B by free-hand technique and only 8 screws were displaced (4 medial perforation and 4 inferior perforation) with the accuracy of screw placement as high as 92.5% (Table 2). The accuracy of screw placement was comparable between left side and right side (L:94.3% (50/53) vs. R:90.6% (48/53), *P* > 0.05). No perforation was larger than 2 mm (grade 1) and no screw-related complication happened (Table 2).

**Table 1:** Comparison of CT Parameters between Trajectory A and Trajectory B

		Trajectory A	Trajectory B	P value
Transverse angle (°)	Right	32.0 ±4.6	14.4 ± 4.1	< 0.001
	Left	33.6 ± 5.5	15.3 ±4.1	< 0.001
Sagittal angle (°)	Right	32.8 ± 8.0	32.5 ± 8.1	0.863
	Left	32.4 ±8.4	32.7 ± 8.1	0.845
Screw length (mm)	Right	32.1 ±3.3	24.7 ± 2.1	< 0.001
	Left	32.9 ±3.7	25.5 ±2.6	< 0.001
Screw width (mm)	Right	5.5 ±1.0	4.4 ±0.7	< 0.001
	Left	5.5 ±1.1	4.6 ±0.9	< 0.001
Sagittal height (mm)	Right	5.5 ±1.0	5.5 ±0.9	0.433
	Left	5.6 ±1.0	5.4 ±0.9	0.252

**Table 2:** Comparison of screw accuracy between left side and right side

	Non-perforation	Perforation	P value
Left side	50	3	0.716
Right side	48	5	

**Table 3:** The medial angulation at C3-7 levels

	Medial angulation (Transverse angle)	
Pedicle	Left	Right
C3	45.8 ± 4.3°	45.0 ± 5.2°
C4	45.2 ± 4.6°	45.6 ± 3.9°
C5	43.4 ± 4.7°	42.9 ± 4.2°
C6	39.2 ± 5.2°	38.9 ± 3.5°
C7	33.6 ± 5.5°	32.0 ± 4.6°

**Table 4:** The existing C7 pedicle screw trajectories in literature

Study	Year	Sample size	Transverse angle	Sagittal angle
By Raj D. Rao, et al.	2008	98	33.5 ± 5.6°	-2.8 ± 3.8°
Xiujun Zheng, et al.	2009	6	34.0 ± 4.0°	-
Dong Ho Lee, et al.	2010	40	28.0 ± 4.6°	-2.0 ± 5.4°
Shaunak Desai, et al.	2010	10	27.1 ± 0.9°	-
Woo Young Jang, et al.	2011	120	35.1 ± 8.1°	-
Wensheng Liao, et al.	2015	6	41.1 ± 1.9°	-
Michael Siu Hei Tse, et al.	2016	94	29.4 ± 3.6°	-
Jarupon Mahiphot, et al.	2019	130	39.4 ± 5.0°	-5.6 ± 4.3°
Manuel Moser, et al.	2019	4	33.4 ± 2.3°	-

## Discussion

Until now, placement of pedicle screw in subaxial cervical spine is still one of the most challenging procedures in spine surgery due to both the small size of cervical pedicle and the proximity of screw trajectory to the vertebral artery or spinal cord [8, 9, 8]. Pedicle screw misplacement in C3 to C6 might lead to catastrophic consequences, which limits the wide application of pedicle screw in these levels [15, 16]. With the development of techniques for O-arm navigation based surgery or robot-assisted surgery, high accuracy of cervical pedicle screw placement was obtained in recent years. Keiji et al. reported a total displacement rate of 3.8% (12/317) in 64 patients with the application of O-arm-based 3D navigation [17]. Sourabh et al. also reported an overall breach rates of only 7.05% after the analysis of cervical pedicle screw insertion by O-arm-based intra-operative navigation [18]. They found that this technique can increase the operator’s confidence in using cervical pedicle instrumentation. Stanley et al. insisted that robotic-guided cervical pedicle screw placement was feasible and safe. In their study, there were only fourteen pedicle screw breaches (15.9%) with deviation no more than 1 mm [19]. Although navigation assistance and robotics have been developed to increase the accuracy of cervical pedicle screw placements, these techniques are only available in a few hospitals in developed countries or regions. In most hospitals, LMS is still the first choice for cervical fixation in C3 to C6. However, the C7 had thicker lateral mass when compared with C3 to C6 and most of C7s had no vertebral artery passing through the transverse foramens [12, 13]. Such unique anatomic characteristics made free-hand technique of pedicle screw safe and feasible for C7 fixation.

Several entry points and trajectories for C7 pedicle screw insertion had been recommended in the literature. Abumi et al. firstly reported the technique for C3-C7 pedicle screws in 1994 [20]. The entry point was located lateral to the center of the articular mass and adjacent to the posterior edge of the superior articular surface with convergent angle of 30–40°. In 1997, Ebraheim et al. introduced a horizontal line between left and right inferior articular processes of upper cervical vertebrae and an ordinate between the outer edge of the lateral mass of the adjacent vertebrae [21]. The entry points were located 1.6–2.6 mm below the horizontal line and 4.5–6.4 mm inward from the ordinate at C3 to C7 levels. In theory, both entry point and convergent angle of C7 should be different from those at other levels since C7 has unique anatomic characteristics as a transitional vertebra. In our study, we also measured the convergent angles of pedicle screws from C3 to C6 (Table 3) and found that the convergent angle at C7 was smaller than those from C3 to C6. Unfortunately, both Abumi et al. and Ebraheim et al. treated C3–C7 as a whole and did not differentiate the entry points for these levels.

Nowadays, there were an increasing number of studies specifically focus on the techniques for C7 pedicle screw insertion. Karaikovic et al. reported different entry points at different cervical levels [15]. He suggested that the entry point was located at the lateral vertebral notch at C3 and C4, but gradually moved medially at C5–C7. However, such description of entry point was too vague for other surgeons to reproduce. Li et al. chose the entry point for C7 pedicle screw as the intersection of the horizontal line through the midpoint of the transverse process root and the vertical line through the intersection of the posterolateral and posterior planes of the isthmus [22]. The screw direction should incline inward by about 60° using this point. Lee et al. recommended a starting point for the C7 pedicle screw to be 2 mm lateral and 2 mm superior to the center of lateral mass with average transverse angles of 28° at C7 [23]. Liao et al. introduced a line connecting point A (the intersection point of the superior margin of the lamina of C7 and the medial margin of the superior articular process) and point B (the intersection point of the lateral margin of the inferior articular process and the transverse process) [24]. The junction site of the middle 1/3 and outer 1/3 segment of this line was selected as the entry point for C7 pedicle screw. The average inclination angle of the screw trajectory was 41.1°.

It is noticeable that most of these recommended entry points for C7 screw were located laterally to the middle of lateral mass, which was often used as the entry points for LMS. In our study, the projection of C7 pedicle axis on lateral mass (Ep A) was also located lateral to the middle of posterior part of C7 with average horizontal offset of 3.7 mm from Ep B no the left side and 3.6 mm on the right side. Selecting these entry points for C7 pedicle screw placement often made rod insertion difficult when the LMS was used at C3 to C6 levels. In addition, a more lateral entry point led to a larger transverse angle for C7 pedicle screw, which increased the difficulty of accurate screw insertion. The average transverse angle of trajectory A was 33.6° on the left side and 32.0° on the right side which was similar to the results of previous studies. Actually, the trajectory A was similar to the C7 pedicle screw trajectories recommended in the literature, which passed through the center of C7 pedicle (Table 4). In the current study, we introduced a easily identified entry point (Ep B) for C7 pedicle screw. This point was kept in line with the entry points for LMS in C3 to C6, which facilitated the procedure of rod insertion (Fig. 5). After exposure of the posterior elements of C7, the base of superior facet is clearly visible and the middle of the base can be quickly identified. The average vertical distance between Ep A and Ep B was only − 0.7 mm on the left side and − 0.8 mm on the right side, which means these 2 entry points were approximately located at the same height in the sagittal plane. However, the, Ep B was located medially to Ep A. The transverse angle should be smaller for pedicle screw trajectory with a more medial entry point. The average transverse angle for trajectory B was only 15.3° on the left side and 14.4° on the right side, which was significantly smaller than that for trajectory A. Due to the small transverse angle, trajectory B can be easily explored by free-hand technique. Although the average screw length for trajectory B was shorter than that for trajectory A, no screw length was smaller than 22 mm for trajectory B.

As we know, intraoperative X-ray film of cervicothoracic junction can not be clearly visualized due to the overlap of the shoulder joint. Therefore, fluoroscopic guidance is not a reliable method for C7 pedicle screw insertion. A safe free hand technique for C7 pedicle screw insertion is necessary for CSM patients underwent posterior cervical fixation. To validate the feasibility of our free hand technique, 53 CSM patients underwent C7 pedicle screw fixation by our technique and the overall accuracy of screw insertion was as high as 92.5% (98/106) with only 8 screws mildly perforated (grade 1). Among these 8 screws, 4 was medially displaced and 4 was downwards displaced. The accuracy of screw placement was similar between left side and right side (L:94.3% vs. R:90.6%, *P* > 0.05). No perforation was larger than 2 mm and no screw-related complication occurred. Actually, all these mild displacements of the C7 screws happened in the early stage of applying this technique. However, this technique is easy for the beginners to learn and finally high accuracy of C7 pedicle screw placement was obtained. Therefore, our method is worthy of being widely popularized. However, we admit that if congenital malformation of C7 was detected in preoperative CT image, intraoperative navigation or robor -assisted instrumentation should be used to ensure accurate C7 pedicle screw placement.

## Data Availability

The datasets used and analysed during the current study available from the corresponding author on reasonable request.
